# Pathological Features of Mitochondrial Ultrastructure Predict Susceptibility to Post-TIPS Hepatic Encephalopathy

**DOI:** 10.1155/2018/4671590

**Published:** 2018-07-16

**Authors:** Hong-bin Li, Zhen-dong Yue, Hong-wei Zhao, Lei Wang, Zhen-hua Fan, Fu-liang He, Xiao-qun Dong, Fu-quan Liu

**Affiliations:** ^1^Department of Interventional Therapy, Beijing Shijitan Hospital, Capital Medical University, China; ^2^Department of Medicine, The Warren Alpert Medical School of Brown University, Providence, RI, USA

## Abstract

**Background:**

Post-TIPS hepatic encephalopathy (PSE) is a complex process involving numerous risk factors; the root cause is unclear, but an elevation of blood ammonia due to portosystemic shunt and metabolic disorders in hepatocytes has been proposed as an important risk factor.

**Aims:**

The aim of this study was to investigate the impact of pathological features of mitochondrial ultrastructure on PSE via transjugular liver biopsy at TIPS implantation.

**Methods:**

We evaluated the pathological damage of mitochondrial ultrastructure on recruited patients by the Flameng classification system. A score ≤2 (no or low damage) was defined as group A, and a score >2 (high damage level) was defined as group B; routine follow-up was required at 1 and 2 years; the incidence of PSE and multiple clinical data were recorded.

**Results:**

A total of 78 cases in group A and 42 in group B completed the study. The incidence of PSE after 1 and 2 years in group B (35.7% and 45.2%, respectively) was significantly higher than that in group A (16.7% and 24.4%, respectively); the 1- and 2-year OR (95% CI) were 2.778 (1.166-6.615) and 2.565 (1.155-5.696), respectively, for groups A and B. Importantly, group B had worse incidence of PSE than group A [P=0.014, hazard ratio (95%CI): 2.172 (1.190-4.678)].

**Conclusion:**

Aggressive damage to mitochondrial ultrastructure in liver shunt predicts susceptibility to PSE. The registration number is NCT02540382.

## 1. Introduction

Transjugular intrahepatic portosystemic shunt (TIPS) is an effective and safe approach for treating esophageal gastric varices bleeding, refractory ascites, and other related complications mainly due to liver cirrhosis and portal vein hypertension. TIPS is currently widely used in clinical practice [1–3]. In addition, problems with the rate of shunt restenosis have decreased dramatically because of the use of covered stents [4–6]; however, another challenge still exists, which is a relatively high (15~48%) incidence of postoperative hepatic encephalopathy [1, 7, 8]. Post-TIPS hepatic encephalopathy (PSE) significantly reduces the curative rate of TIPS, quality of postoperative life, and overall survival of patients [3, 9]. Presently, PSE remains a major challenge in clinical practice.

PSE is a complex process that involves numerous factors interacting with one another, which may be related to patient age, stent diameter, preoperative liver function, preoperative and postoperative portal vein blood flow direction, pressure, sarcopenia, and the presence of preoperative hepatic encephalopathy [10–12]. Moreover, Silvia Nardelli et al. performed a prospective study that indicated sarcopenia as a risk factor independently associated with development of PSE, which may be related to the reduction of the capacity of ammonia removal[13]. Elevation of blood ammonia due to portosystemic shunt has been proposed as a risk factor, mainly because toxic ingredients in portal vein blood can openly access the systemic circulation directly after the shunt without being first detoxified by the liver, thus promoting the development of hepatic encephalopathy [14–16]. In fact, increased systemic blood ammonia partially enters the liver through the hepatic artery [17, 18]. In addition, hepatic hemodynamic changes after TIPS aggravate metabolic disorders [15, 19, 20]. Therefore, systemic blood ammonia at varying levels after TIPS may be related to the degree of blood ammonia metabolic disorders that occur in hepatic cellular mitochondria. However, whether the occurrence of PSE results from ammonia metabolic disorders due to mitochondrial damage and whether the pathological features of mitochondrial ultrastructure can be used as a biomarker to assess mitochondrial function remain unknown [21–24]. In the current study, transjugular liver biopsy was obtained during positioning of the TIPS, and a transmission electron microscope was used to observe the pathological features of mitochondrial ultrastructure to fill in knowledge gaps. A semiquantitative scoring method was adopted to evaluate mitochondrial damage according to the Flameng classification system. The relationship between the pathological features of mitochondria ultrastructure and the incidence of PSE was explored in 150 patients recruited from January 2012 to December 2013.

## 2. Materials and Methods

### 2.1. Patient Selection

In total, 150 patients who underwent TIPS at Beijing Shijitan Hospital from January 2012 to December 2013 were recruited. The inclusion criteria were confirmed diagnosis of posthepatic cirrhosis portal hypertension; scheduled for elective TIPS; transjugular liver biopsy obtained during positioning of the TIPS successfully; and shunt channel with a diameter of 8 mm. The exclusion criteria were aged <18 or >70 years; combined with malignant liver tumor; liver tissue not conforming with the requirements (the size of liver tissue is < 0.4×0.4 cm^2^); and hepatic encephalopathy before TIPS.

### 2.2. Methods

Transjugular liver biopsy was obtained during positioning of the TIPS and a transmission electron microscope was adopted to identify the pathological features of mitochondrial ultrastructure; thus, patients were prospectively divided into subgroups according to the level of mitochondrial damage for follow-up observation.

#### 2.2.1. TIPS and Obtaining Liver Tissue from Preshunt Channel

**Figure 1:**
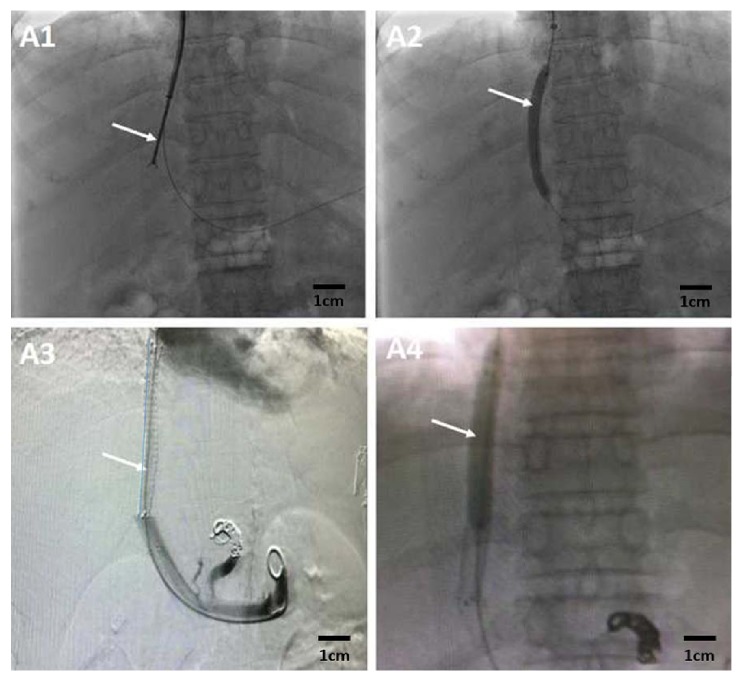
Procedure for obtaining liver tissue of preshunt channel. A1: A RUPS-100 liver access set was delivered into the portal vein. Liver tissue of the shunt was obtained before balloon dilation (white arrow). A2: Balloon dilation of the shunt (white arrow). A3: Stent was implanted among the inferior vena cava, hepatic vein, preshunt channel, and portal vein to establish a shunt (white arrow). A4: Balloon dilation of the stent (white arrow).

Local disinfection and anesthesia were performed at the selected piercing site, and then jugular vein puncture was conducted. A liver access set (RUPS-100; Cook, USA) was delivered into the hepatic vein or hepatic inferior vena cava, the left and right trunk of the portal vein or portal vein bifurcation were punctured, and the liver access set was then placed in the portal vein. A pigtail catheter was used for portography and measurement of portal venous pressure. The leading end of the ultra-smooth ultra-long hard guide wire was placed in the superior mesenteric vein or splenic vein. The sheath of the liver access set was withdrawn into the preshunt channel in the liver parenchyma, and biopsy forceps (Minimally Invasive Medical Technology Co., LTD, Nanjing, China) were inserted through the sheath to obtain liver tissue of the preshunt channel in the liver parenchyma (the size range of each obtained liver tissue sample ranged from 0.4×0.4 cm^2^ to 0.8×0.8 cm^2^, and recollection of tissue was required for samples of a smaller size) (Figure 1-A1). A balloon was introduced along the guide wire to dilate the shunt, and then a covered stent (Bard Fluency) with a diameter of 8 mm was implanted (Figures 1-A2 and 1-A3), followed by stent dilation (Figure 1-A4), measurement of portal venous pressure, and portography.

#### 2.2.2. Postoperative Routine Observation and Treatment

All patients were asked to stay in bed for 24 hours after the operation; pressure dressing and sand bag pressing were applied to the piercing site area, and the vital signs of each patient were monitored in real time. Prophylactic antibiotics were employed. Subcutaneous injection of low molecular weight heparin (5000 IU, Bid) was performed from the second day after the operation and lasted for at least 5 days; then treatment was switched to an oral intake of warfarin for at least half a year (2.5-5.0 mg, Qd). The dose of warfarin was adjusted based on coagulation function every 15 days to ensure an INR between 2 and 3. Oral administration of branched chain amino acids (3 g, Tid) and lactulose (15-30 ml, Bid or Tid) was performed routinely to prevent hepatic encephalopathy. A liver protection strategy was also taken (bicyclol tablets, 25 mg, Tid).

#### 2.2.3. Observing Pathological Features of Mitochondrial Ultrastructure on Transmission Electron Microscopy (TEM)

**Figure 2:**
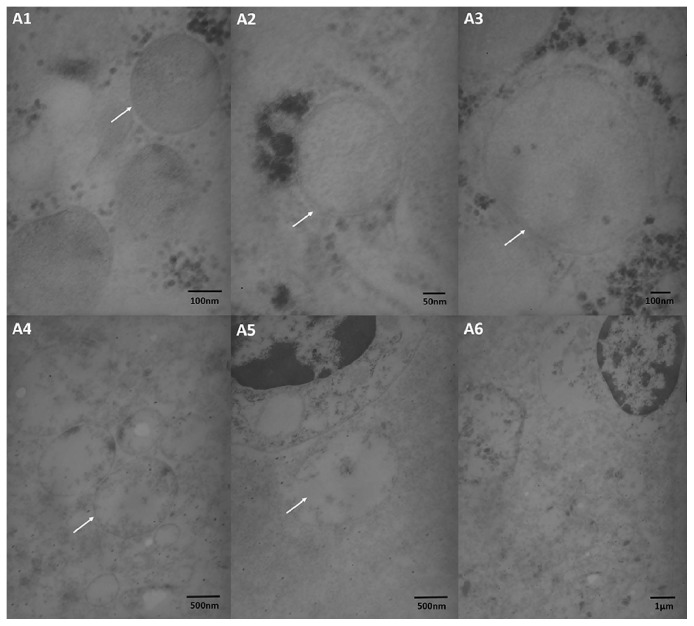
Mitochondrial ultrastructure at 5 different damage levels according to the Flameng classification system. A1: Level 0 (score 0) mitochondrial structure is normal and full of substrate particles (white arrow). A2: Level 1 (score 1) mitochondrial structure is essentially normal with a lack of substrate particles (white arrow). A3: Level 2 (score 2) mitochondria are markedly swollen with transparent substrate (white arrow). A4: Level 3 (score 3) mitochondrial crest is divided with transparent or thick substrate (white arrow). A5: Level 4 (score 4) mitochondria are vacuolated with divided crest, and substrate and membrane integrity have disappeared (white arrow). A6: microcellular ultrastructure vision obtained more mitochondrial structure.

Liver tissue obtained during TIPS was directly transferred into 3% glutaraldehyde, fixed with 1% osmium tetroxide, dehydrated in ethanol solution, infiltrated in a mixed solution of Epon-812 agar and acetone, embedded overnight, and then polymerized in pure Epon-812 agar. The polymerized blocks were stained by toluidine blue, embedded, and sectioned with LKB-V ultramicrotome successively. Subsequently, the pathological features of mitochondrial ultrastructure were observed in the samples, and photos were taken by transmission electron microscopy (TEM, HITACHIH-600). Five fields were randomly selected in the electron microscopy images of each specimen, and then at least 20 mitochondria were randomly selected in each field to obtain a semiquantitative score of mitochondria according to the Flameng classification system [25]. Mitochondria were graded with scores of 0-4 according to the degree of damage, with a higher score representing a higher degree of damage. The damage of each mitochondrion and the average score for all mitochondria were evaluated independently by at least two investigators to avoid bias (Figure 2).

#### 2.2.4. Recording Multiple Preoperative and Intraoperative Clinical Characteristics of Patients

Preoperative baseline data included age, gender, CTP, MELD score, AST, ALT, and venous blood ammonia level. Intraoperative data included portal pressure gradient (PPG) before and after shunt and PPG reduction.

#### 2.2.5. Cohort Formation

On the basis of semiquantitative scores, liver tissue obtained from the shunts was evaluated according to the Flameng classification system. Accordingly, patients were divided into 2 groups: a score ≤2 (no or low level of damage) was defined as group A, whereas a score >2 (higher level of damage) was defined as group B. All cases were followed up for up to 2 years.

#### 2.2.6. Follow-Up

Routine follow-up was required for all patients before discharge and at 3 months, 6 months, 12 months, 18 months, and 24 months after TIPS. Medical history, physical examination, laboratory tests (e.g., routine blood, liver and kidney function, blood coagulation, and venous blood ammonia), abdominal CT/MRI, portal venous ultrasound, and endoscopy were conducted at each time point. In particular, venous blood ammonia level and incidence of PSE were recorded. PSE was assessed and graded on admission by a single investigator and confirmed by a senior investigator using the West Haven Criteria for grading of mental status[26].

### 2.3. Statistical Analysis

Statistical analysis was conducted using SPSS software (version 22.0). Quantitative data were described as the mean ± standard deviation (SD) and compared by independent sample* t*-test. Qualitative data were compared by *χ*^2^ test or Fisher's exact test. Pearson correlation analysis was used to correlate two continuous variables. Hepatic encephalopathy cumulative risk was estimated using a Kaplan-Meier plot, and Log-rank (Mantel-Cox) test was used to calculate the hazard ratio. A* p* value of <0.05 was considered statistically significant.

### 2.4. Ethical Requirements and Informed Consent

This study had been approved by the Institutional Review Board (IRB) Committee in Beijing Shijitan Hospital, Capital Medical University. Informed consent was acquired from each participant before the operation. All procedures were conducted according to the guidelines approved by the Ethics Committee in Beijing Shijitan Hospital, Capital Medical University.

## 3. Results

### 3.1. Patient Population

**Figure 3:**
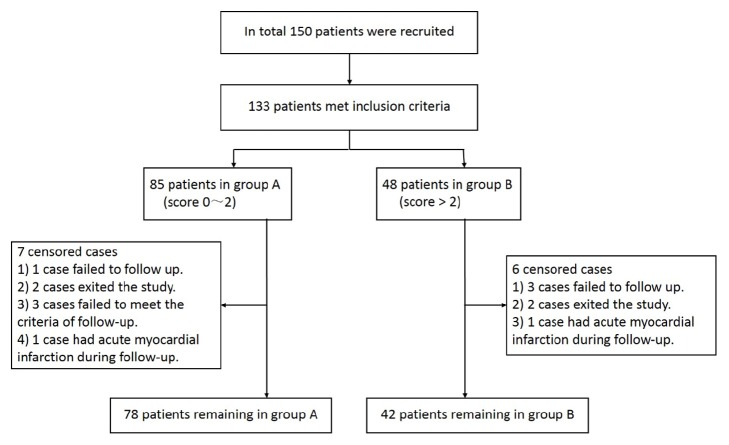
Flowchart of patient recruitment and selection.

A total of 133 patients met the inclusion criteria and were recruited from January 2012 to December 2013; however, 13 patients were excluded because of failure to meet the criteria during the operation or follow-up. Finally, 120 patients (78 cases in group A and 42 cases in group B) completed the study (Figure 3). No procedure-related deaths or serious complications (e.g., abdominal bleeding, hepatic failure, or distant embolism) occurred. Clinical symptoms improved to varying degrees.

### 3.2. Perioperative Patient Information

Perioperative clinical characteristics of the two groups were compared. No significant differences were observed in age, gender, Child-Pugh stage, MELD score, AST, ALT, preoperative blood ammonia level, or PPG before or after shunt between the two groups (Table 1).

### 3.3. Relationship between Hepatic Encephalopathy and Mitochondrial Damage

**Figure 4:**
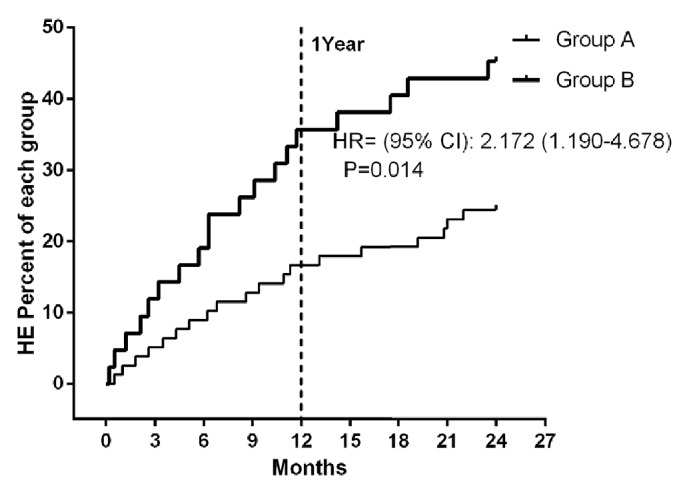
HE cumulative risk was estimated by Kaplan-Meier curves. Log-rank (Mantel-Cox) test was used to calculate the hazard ratio. Compared to group A, group B had a significantly increased risk of HE during the study (p<0.05).

The incidence of HE 1 year after TIPS was notably higher in group B (13/42, 35.7%) than in group A (15/78, 16.7%) with an OR (95% CI) of 2.778 (1.166-6.615). The incidence of HE 2 years after TIPS was significantly higher in group B (19/42, 45.2%) than in group A (19/78, 24.4%) with an OR (95% CI) of 2.565 (1.155-5.696) (Table 2), except for 1 case of group B for grade II, and the rest are grade I. In addition, univariate competing risk regression for time to PSE is reported in Table 3. At multivariate analysis, ammonia (HR 1.830, 95%CI 1.093-3.712, p=0.032) and mitochondrial damage level (HR 2.172, 95%CI 1.190-4.678, p=0.014) were independently associated with PSE development, which showed an induced risk of PSE in group B compared to that in group A (Figure 4).

### 3.4. Relationship between Postoperative Blood Ammonia Level and Mitochondrial Damage

For follow-up of up to 1 year (before discharge, 3 months, 6 months, and 12 months), the average venous blood ammonia level (*μ*mol/L) in group A was significantly lower than that in group B (64.2±15.7 and 95.8±21.4, respectively). For follow-up of up to 2 years (before discharge, 3 months, 6 months, 12 months, 18 months, and 24 months), the average venous blood ammonia level (*μ*mol/L) in group A was significantly lower than that in group B (53.4±16.5 and 83.3±18.9, respectively) (Table 4).

### 3.5. Relationship between Average Blood Ammonia Level and PSE

At 1-year follow-up, the average venous blood ammonia level of 28 patients with PSE was 110.8±18.7 *μ*mol/L and that of 92 patients without PSE was significantly lower at 55.8±21.1 *μ*mol/L (*P*<0.005). At 2-year follow-up, the average venous blood ammonia level of 38 patients with PSE was 99.4±20.9 *μ*mol/L and that of 82 patients without PSE was significantly lower at 51.6±16.7 *μ*mol/L (*P*<0.013) (Table 5), At multivariate analysis, ammonia (HR 1.830, 95%CI 1.093-3.712, p=0.032 ) was independently associated with PSE development.

### 3.6. Correlation between Average Ammonia Level and Flameng Classification Scores

Pearson correlation analysis demonstrated that the average postoperative venous blood ammonia level and mitochondrial Flameng classification scores were positively correlated. The correlation coefficients at 1 year and 2 years were r=0.574 (P=0.040) and r=0.531 (P=0.017), respectively.

## 4. Discussion

In cirrhosis, liver ischemia hypoxia, endotoxin, inflammatory reaction, and immune factors can cause mitochondrial or cytoplasmic damage [27–29]. Dysfunction of mitochondrial respiratory chain complex III causes damage to the electron transport chain, and then more reactive oxygen species (ROS) are generated as substrates. ROS induce mitochondrial lipid peroxidation reactions to produce malondialdehyde (MDA), which triggers mitochondrial membrane permeability transition by activating mitochondrial permeability transition pore (mPTP) and calcium dyshomeostasis and further undermines mitochondrial oxidative phosphorylation as a vicious circle [30–33], thus resulting in tricarboxylic acid cycle (TCA) disturbance. The urea cycle and TCA are correlated and interdependent. NH_3_ entering liver cells requires the TCA to provide enough energy to activate and maintain a series of enzymatic reactions in mitochondria and the cytoplasm. Carbamoyl phosphate synthetase I (CPS-I) and ornithine carbamoyl transferase (OCT) are two of the most important enzymes in the urea cycle, and CPS-I is the rate-limiting enzyme. The levels of CPS-I and OCT can reflect mitochondrial enzymatic activity and urea cycle function in hepatocytes [34, 35]. The ability of mitochondria to transform blood ammonia can be weakened to varying degrees because of decreased levels of CPS-I when liver function is seriously damaged, leading to increased blood ammonia and hepatic encephalopathy [36, 37].

In addition, alterations in nutritional status are frequently associated with liver disease. Sarcopenia, a condition of loss of muscle mass, is associated with cirrhosis' complications, including HE. Nardelli S et al.[13] designed a study to investigate whether a decrease in muscle mass was independently associated with the occurrence of PSE. The results (sarcopenia HR, 31.3; 95% CI, 4.5–218.07; P<0.001) support the viewpoint that sarcopenia is risk factor for development of PSE; the rationale for this relationship derives from the possible involvement of muscle in ammonia metabolism and trafficking. This study suggests that sarcopenia should be considered in selecting the patients for TIPS therapy. Nutritional status should be evaluated in patients with sarcopenia before TIPS placement, which might reduce the incidence of PSE. Considering the results of this study, the indices of sarcopenia in the examined patients that should be incorporated into our study could be a possible bias of our work.

On the other hand, a prospective study [38] found that early decreasing ammonia obviously improved the survival rate and prognosis of patients with liver failure. It is speculated that decreasing the blood ammonia level effectively attenuates mitochondrial damage and promotes the functional recovery of hepatocellular detoxification, synthesis, and transformation. Meanwhile, elevated blood ammonia may cause a “secondary damage” to hepatocellular mitochondria as an alternative indicator. Yu et al. established a rat model with acute high blood ammonia attack and found that high blood ammonia aggravated liver failure through “secondary injury” [39, 40]. Presently, the underlying mechanism remains unclear. One possibility is that ammonia accumulated in hepatocytes is initially transported by regulating ammonia transport-related proteins AQP8 and RHCG in mitochondria, which impairs the structure and function of mitochondria via opening mPTP and the intrinsic apoptotic pathway, thus causing energy metabolic disorders and oxidative damage, which affect the urea cycle [41–43].

In our study, the incidence of PSE in group B was approximately doubled (2.14- and 1.85-fold at 1 and 2 years, respectively) compared to that in group A. As an independent risk factor, the average blood ammonia level of PSE cases was almost doubled compared to that of non-PSE cases at 1 year and 2 years. Our results support the viewpoint that elevated postoperative blood ammonia correlates with hepatic encephalopathy [44, 45].

Notably, postoperative average venous blood ammonia levels and mitochondrial Flameng classification scores were positively correlated at 1 year and 2 years. In addition, HE cumulative risk was significantly increased in group B, which had a higher level of mitochondrial damage. Our results indicate that mitochondrial damage predicts blood ammonia metabolic disorders and subsequently positively correlates with risks of PSE; elevated blood ammonia may serve as an important bridge.

## 5. Conclusion

In summary, the pathological features of mitochondrial ultrastructure in transjugular liver biopsy positively correlate with increased postoperative average blood ammonia and, more importantly, the development of PSE. Therefore, this approach could be important for helping evaluate the reserve of liver ammonia metabolism as demonstrated by mitochondrial damage, which is of great significance for forecasting and preventing HE. However, this preliminary research is limited to partial mitochondrial damage of liver shunt and funds; therefore, further in-depth investigations (such as studies including other metabolic derangement parameters, e.g., ROS, RCR, MAPR, and membrane potential) are required as is a larger sample size.

## Figures and Tables

**Table 1:** Perioperative clinical characteristics of two groups.

Items	Group A	Group B	*t/χ* ^*2*^	*P*
Patient (n)	78	42		
Gender (M/F, n)	54/24	30/12	0.063	0.802
Age (mean ± SD, years)	54.2±8.18	52.9±9.13	0.775	0.440
CTP (n, %)			6.753	0.056
Stage A	39 (50.0)	25 (59.5)		
Stage B	24 (30.8)	13 (31.0)		
Stage C	15 (19.2)	4 (9.5)		
MELD score	7.04±3.21	8.23±4.83	1.250	0.214
ALT (U/L)	28.6±14.4	28.0±15.4	0.215	0.830
AST (U/L)	47.7±12.6	45.3±13.2	0.342	0.647
Ammonia (mean ± SD, *μ*mol/L)	50.4±17.0	64.8±29.3	1.741	0.306
PPG (mean ± SD, mmHg)				
Before shunt	26.0±4.67	25.7±4.82	0.431	0.668
After shunt	15.1±4.31	15.2±4.10	0.030	0.976
PPG reduction (mean ± SD, mmHg)	10.9±2.60	10.5±2.51	0.841	0.402

ALT: alanine transaminase; AST: aspartate aminotransferase; CTP: Child-Pugh stage; MELD: model for end-stage liver disease; PPG: portal pressure gradient.

**Table 2:** Incidence of HE stratified by groups.

PSE incidence	Group A	Group B	OR (95% CI)	*P*
1-year (n, %)	13 (16.7)	15 (35.7)	2.778 (1.166-6.615)	0.019
2-year (n, %)	19 (24.4)	19 (45.2)	2.565 (1.155-5.696)	0.019

PSE: post-TIPS hepatic encephalopathy; OR: odds ratio.

**Table 3:** Univariate competing risk regression for the relationship between clinical characteristics of the patients and time to PSE.

Items	PSE Present (n=38)	PSE Absent (n=82)	HR(95%CI)	*P*
Age (mean ± SD, years)	55.3±8.31	51.9±8.97	0.634(0.218-1.402)	NS
CTP Stage A/B/C (n.)	19/14/5	45/23/14	1.482(0.547-3.563)	NS
MELD score	8.07±3.96	7.36±4.13	0.744(0.156-1.215)	NS
Ammonia (mean ± SD, *μ*mol/L)	51.6±16.7	99.4±20.9	1.997(1.163-3.563)	0.027
PPG reduction (mean ± SD, mmHg)	11.3±1.51	10.1±2.63	1.133(0.896-1.427)	NS
Mitochondrial damage(Group A/B)	19/19	23/59	2.561(1.615-3.873)	0.017

HR: hazard ratio.

**Table 4:** Postoperative average ammonia levels stratified by groups.

Index	Group A	Group B	*t*	*P*
1-year Ammonia (mean ± SD, *μ*mol/L)	64.2±15.7	95.8±21.4	3.733	0.002
2-year Ammonia (mean ± SD, *μ*mol/L)	53.4±16.5	83.3±18.9	3.279	0.003

**Table 5:** Average blood ammonia level and PSE.

PSE	Absent	Present	*t*	*P* ^*∗*^
1-year Ammonia (mean ± SD, *μ*mol/L)	110.8±18.7	55.8±21.1	3.216	0.005
2-year Ammonia (mean ± SD, *μ*mol/L)	99.4±20.9	51.6±16.7	2.423	0.013

## Abbreviations

ALT: Alanine transaminase
AST: Aspartate aminotransferase
CTP: Child-Pugh stage
CPS-I: Carbamoyl phosphate synthetase I
HR: Hazard ratio
MELD: Model for end-stage liver disease
MDA: Malondialdehyde
mPTP: Mitochondrial permeability transition pore
MAPR: Mitochondrial ATP production rate
OR: Odds ratio
OCT: Ornithine carbamoyl transferase
PSE: Post-TIPS hepatic encephalopathy
PPG: Portal pressure gradient
ROS: Reactive oxygen species
RCR: Respiratory control ratio
TIPS: Transjugular intrahepatic portosystemic shunt
TCA: Tricarboxylic acid cycle.
